# Supplementary material for: Feasibility of Arterial Spin Labeling Magnetic Resonance Imaging for Musculoskeletal Tumors with Optimized Post-Labeling Delay
Source: Diagnostics (Basel). 2022 Oct 10;12(10):2450. doi: 10.3390/diagnostics12102450 (PMC9600497; doi:10.3390/diagnostics12102450)
Supplement: Supplementary file 1 [file diagnostics-12-02450-s001.zip › Figure S1 mean TBF values in the patients without spinal artifacts.pdf]

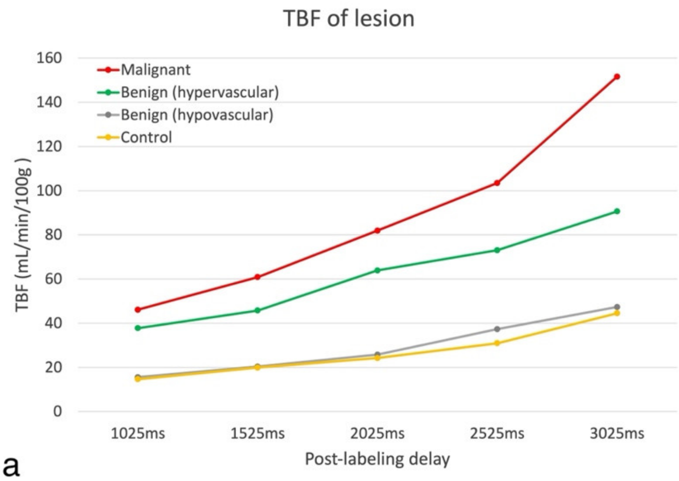
TBF of lesion
Malignant
Benign (hypervascular)
Benign (hypovascular)
Control
TBF (mL/min/100g)
160
140
120
100
80
60
40
20
0
1025ms
1525ms
2025ms
2525ms
3025ms
Post-labeling delay

a

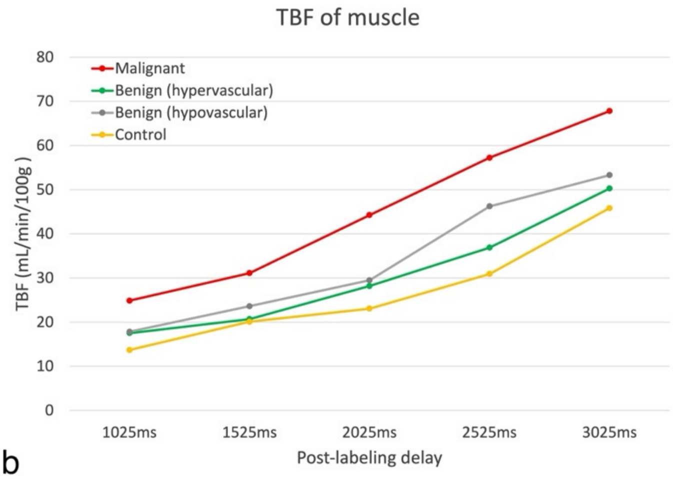
TBF of muscle
Malignant
Benign (hypervascular)
Benign (hypovascular)
Control
TBF (mL/min/100g)
80
70
60
50
40
30
20
10
0
1025ms
1525ms
2025ms
2525ms
3025ms
Post-labeling delay

b

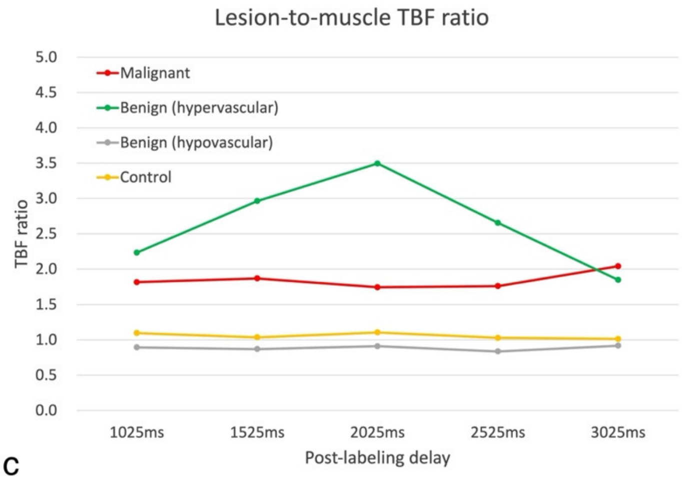
Lesion-to-muscle TBF ratio
Malignant
Benign (hypervascular)
Benign (hypovascular)
Control
TBF ratio
5.0
4.5
4.0
3.5
3.0
2.5
2.0
1.5
1.0
0.5
0.0
1025ms
1525ms
2025ms
2525ms
3025ms
Post-labeling delay

c

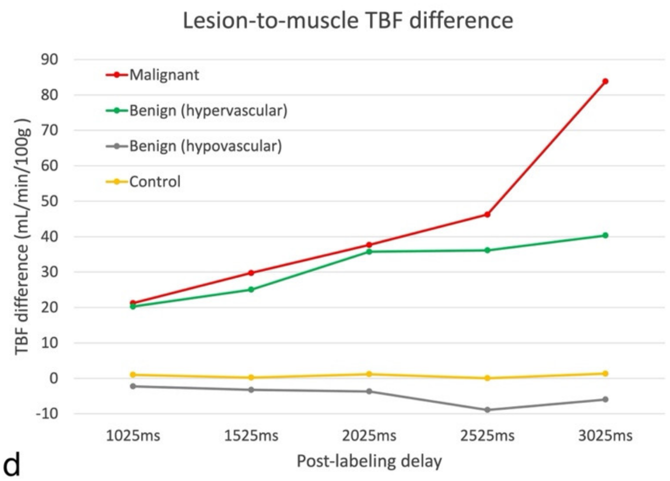

d

Figure S1. The mean TBF values in the patients without spiral artifacts. The mean TBF values of lesions (a) and normal muscles (b), the lesion-to-muscle differences (c) and the lesion-to-muscle TBF ratios (d) of the malignant group, the hypervascular benign group, the hypovascular benign group and the control group in different post-labeling delay time.
