# Supplementary material for: Feasibility of Arterial Spin Labeling Magnetic Resonance Imaging for Musculoskeletal Tumors with Optimized Post-Labeling Delay
Source: Diagnostics (Basel). 2022 Oct 10;12(10):2450. doi: 10.3390/diagnostics12102450 (PMC9600497; doi:10.3390/diagnostics12102450)
Supplement: Supplementary file 1 [file diagnostics-12-02450-s001.zip › Figure S2 relation between TBF values in the patients without spinal artifacts.pdf]

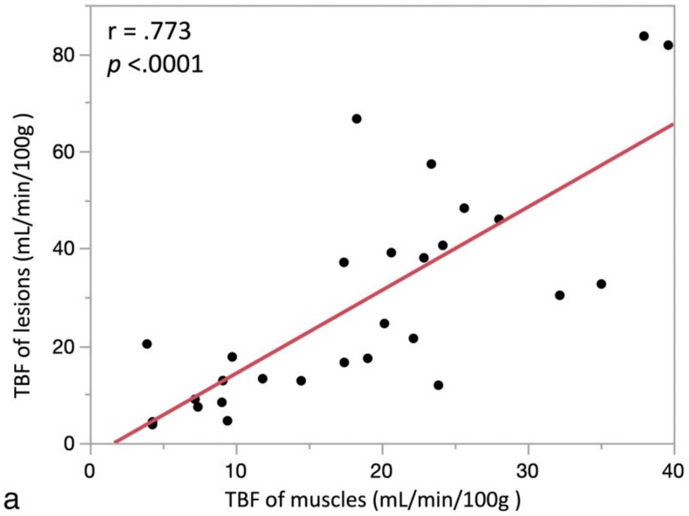
r = .773
p <.0001
TBF of lesions (mL/min/100g)
TBF of muscles (mL/min/100g)
0
20
40
60
80
0
10
20
30
40
a

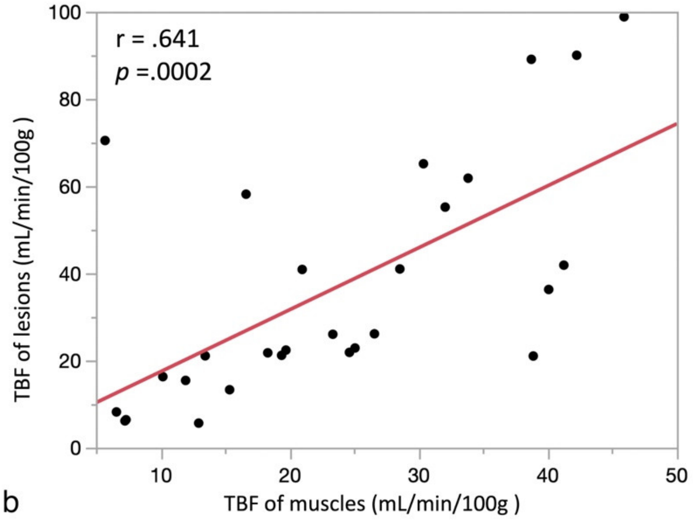
r = .641
p =.0002
TBF of lesions (mL/min/100g)
TBF of muscles (mL/min/100g)
0
20
40
60
80
100
10
20
30
40
50
b

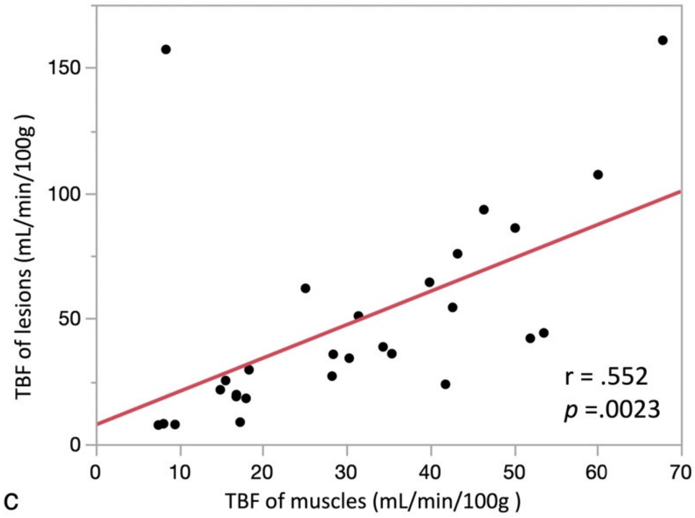
r = .552
p =.0023
TBF of lesions (mL/min/100g)
TBF of muscles (mL/min/100g)
0
50
100
150
0
10
20
30
40
50
60
70
c

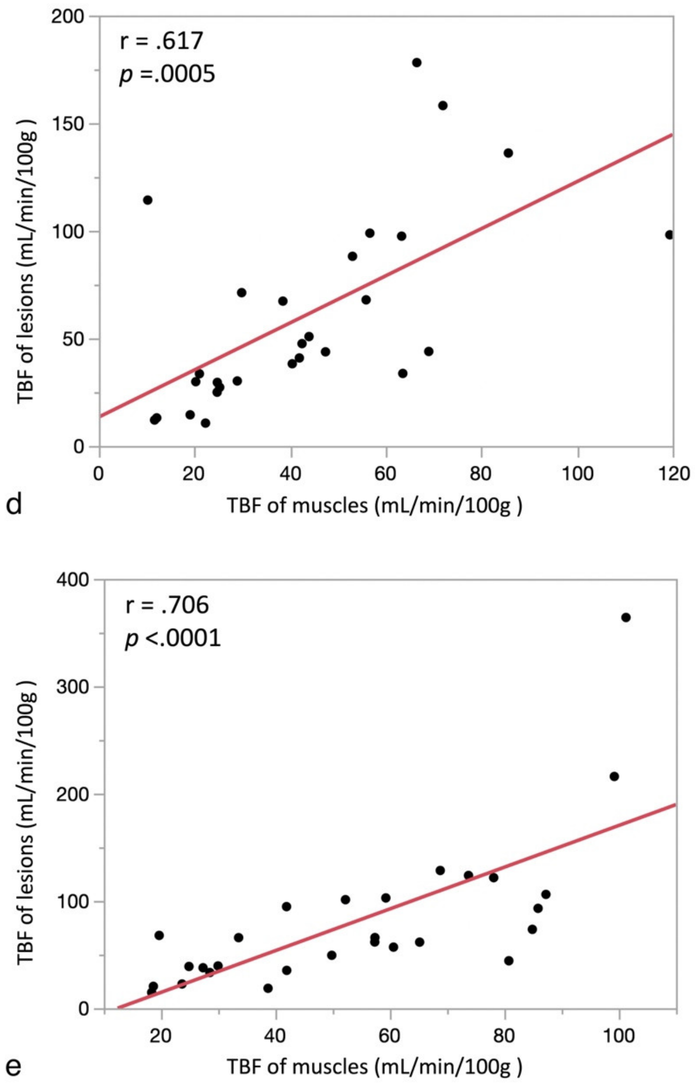

Figure S2. Relation between the mean TBF values of lesions and muscles in patients without spiral artifacts. Relation between the mean TBF values of lesions and muscles in the different PLD groups (a: PLD=1025ms; b: PLD=1525ms; c: PLD=2025ms; d: PLD=2525ms; e: PLD=3025ms).
