# Supplementary material for: Feasibility of Arterial Spin Labeling Magnetic Resonance Imaging for Musculoskeletal Tumors with Optimized Post-Labeling Delay
Source: Diagnostics (Basel). 2022 Oct 10;12(10):2450. doi: 10.3390/diagnostics12102450 (PMC9600497; doi:10.3390/diagnostics12102450)
Supplement: Supplementary file 1 [file diagnostics-12-02450-s001.zip › Table S1 Imaging criteria for diagnosis of the benign lesions.pdf]

Table S1. Imaging criteria for diagnosis of the benign lesions in this study.

Diagnosis	Imaging Diagnosis Criteria
**Soft Tissue Tumors**	
Schwannoma	General features:<br>. Well-defined mass<br>. Fusiform mass eccentrically positioned in relation to parent nerve<br>. Fat-split sign (+)<br>T1:<br>. Homogeneously isointense to slightly hyperintense relative to muscle<br>T2 with fat suppression:<br>. Heterogeneously hyperintense to muscle<br>Post-enhanced T1 with fat suppression:<br>. Heterogeneously intense enhancement
Hemangioma	General features:<br>. Well-defined mass<br>. Maybe lobulated<br>. Commonly have intralesional fat and phleboliths<br>T1:<br>. Isointense or hypointense to muscle on T1-weighted imaging<br>. Intralesional hyperintense component (fat)<br>T2:<br>. Heterogeneously hyperintense on T2-weighted images<br>. Intralesional hypointense nodule (phleboliths)<br>Post-enhanced T1 with fat suppression:<br>. Gradually intense enhancement
Lipoma	General features:<br>. Well-defined mass<br>MRI:<br>. Homogenously hyperintense on non-fat-suppressed images<br>. Homogeneously hypointense on fat-suppressed images<br>. No significant enhancement
Cyst	General features:<br>. Well-defined mass<br>T1:<br>. Homogeneously and significantly hypointense<br>T2:<br>. Homogenously hyperintense<br>Post-enhanced T1 with fat suppression:<br>. No enhancement or mild capsular enhancement
Bone tumors	
Fibrous dysplasia	General features:<br>. Elongated, mildly expansile lesion in long bone<br>. Well-defined lesoin<br>. Variable internal sclerosis depending on the underlying constituents<br>T1:<br>. Homogeneously, isointense to slightly hyperintense relative to muscle<br>T2:<br>. Variable T2 signal changes ranging from hypointense regions to areas of hyperintense signal, including chondroid elements and areas of cystic change<br>. No surrounding marrow edema<br>Post-enhanced T1 with fat suppression:<br>. Enhancement in the areas with high T2-weighted intensity
Enchondroma	General features:<br>. Geographic, well-defined, osteolytic mass

. Lobular morphology.
. Usually with matrix mineralization (calcification)

T1:
. Isointensity to hypointensity relative to muscle
. Intermixed with areas of hypointensity corresponding to matrix mineralization

T2:
. Hyperintensity relative to muscle
. Intermixed with areas of hypointensity corresponding to matrix mineralization
. No surrounding marrow edema

Post-enhanced T1 with fat suppression:
. No significant enhancement or subtle enhancement around calcifications
. May have septal and peripheral (multilobular rim-like) gadolinium enhancement (should rule out chondrosarcoma)
