# Supplementary material for: Feasibility of Arterial Spin Labeling Magnetic Resonance Imaging for Musculoskeletal Tumors with Optimized Post-Labeling Delay
Source: Diagnostics (Basel). 2022 Oct 10;12(10):2450. doi: 10.3390/diagnostics12102450 (PMC9600497; doi:10.3390/diagnostics12102450)
Supplement: Supplementary file 1 [file diagnostics-12-02450-s001.zip › Table S2 Details of pathological diagnosis of the malignant group in the patients without spiral artifacts.pdf]

Table S2. Details of pathological diagnosis of the malignant group in the patients without spiral artifacts.

Diagnosis	N	Location (N)
Atypical lipomatous tumor (borderline malignancy)	1	Left thigh (1)
Liposarcoma, well-differentiated	1	Left thigh (1)
Pleomorphic rhabdomyosarcoma	1	Left thigh (1)
Myxofibrosarcoma	1	Right thigh (1)
Lymphoma	1	Right iliac bone (1)
Metastasis	1	Right iliac bone (1)
