# Supplementary material for: Feasibility of Arterial Spin Labeling Magnetic Resonance Imaging for Musculoskeletal Tumors with Optimized Post-Labeling Delay
Source: Diagnostics (Basel). 2022 Oct 10;12(10):2450. doi: 10.3390/diagnostics12102450 (PMC9600497; doi:10.3390/diagnostics12102450)
Supplement: Supplementary file 1 [file diagnostics-12-02450-s001.zip › Table S3 Details of diagosis of the benign groups in the patients without spiral artifacts.pdf]

Table S3. Details of pathological and imaging diagnosis of the benign groups in the patients without spiral artifacts.

Diagnosis	N	Method	Location (N)
Hypervascular benign lesion	7		
Schwannoma	3	Pathology	Right hip (1)
		MRI	Right leg (2)
Hemangioma	3	Pathology	Right foot (1)
		MRI	Right lower leg (2)
Fibrous dysplasia	1	MRI	Left femur (1)
Hypovascular benign lesion	15		
Intramuscular myxoma	1	Pathology	Right hip (1)
Lipoma	5	Pathology	Right gluteal region (1)
		MRI	Right thigh (2)<br>Left thigh (2)
			Left foot (1)
Cyst	3	MRI	Left knee (1)
			Right ankle (1)
Enchondroma	1	MRI	Right tibia (1)
Necrosis	1	Pathology	Right thigh (1)
Synovial chondromatosis	2	Pathology	Right knee (1)<br>Left knee (1)
Amyloidosis	1	Pathology	Left lower leg (1)
Gouty arthritis	1	Pathology	Left knee (1)
