# Supplementary material for: Feasibility of Arterial Spin Labeling Magnetic Resonance Imaging for Musculoskeletal Tumors with Optimized Post-Labeling Delay
Source: Diagnostics (Basel). 2022 Oct 10;12(10):2450. doi: 10.3390/diagnostics12102450 (PMC9600497; doi:10.3390/diagnostics12102450)
Supplement: Supplementary file 1 [file diagnostics-12-02450-s001.zip › Table S4 TBF and TBF difference and ratio in the patients without spiral artifacts.pdf]

Table S4. TBF and TBF difference and ratio in the patients without spiral artifacts. TBF of lesions and normal muscles and lesion-to-muscle TBF difference and ratio in the post-Labeling delay of 1025ms to 3025 ms and the *p* value between the malignant, hypervascular benign, hypovascular benign and control groups in the patients without spiral artifacts.

	Malignant	Hypervascular Benign	Hypovascular Benign	Control	M-B	M-L	M-N	B-L	B-N	L-N
PLD=1025ms										
Lesion TBF (mL/min/100g)	46.07 ± 22.37	37.75 ± 27.87	15.55 ± 10.12	14.65 ± 9.27	0.8398	0.0201	0.0142	0.0526	0.0357	0.9995
Muscle TBF (mL/min/100g)	24.86 ± 8.32	17.51 ± 11.92	17.82 ± 10.73	13.69 ± 6.86	0.5347	0.5549	0.1650	0.9999	0.8173	0.7654
Dif_TBF (mL/min/100g)	21.21 ± 15.01	20.24 ± 18.14	-2.27 ± 3.85	0.97 ± 6.53	0.9988	0.0041*	0.0135	0.0010*	0.0040*	0.9166
TBF ratio	1.82 ± 0.40	2.23 ± 1.39	0.89 ± 0.23	1.09 ± 0.40	0.7632	0.1450	0.3164	0.0033*	0.0120*	0.9309
PLD=1525ms										
Lesion TBF (mL/min/100g)	60.83 ± 29.69	45.72 ± 31.16	20.36 ± 12.46	19.88 ± 9.74	0.5752	0.0070*	0.0054*	0.0600	0.0471	0.9999
Muscle TBF (mL/min/100g)	31.10 ± 10.27	20.68 ± 13.44	23.60 ± 13.37	20.08 ± 9.49	0.4091	0.6603	0.3308	0.9502	0.9995	0.9046
Dif_TBF (mL/min/100g)	29.73 ± 19.90	25.04 ± 24.66	-3.24 ± 5.87	-0.20 ± 8.19	0.9476	0.0027*	0.0060*	0.0021*	0.0053*	0.9691
TBF ratio	1.87 ± 0.45	2.96 ± 3.60	0.87 ± 0.24	1.03 ± 0.32	0.7147	0.7577	0.8367	0.0856	0.1149	0.9969
PLD=2025ms										
Lesion TBF (mL/min/100g)	81.89 ± 48.39	63.90 ± 48.28	25.76 ± 14.33	24.20 ± 12.20	0.7424	0.0150	0.0105*	0.0629	0.0433	0.9995
Muscle TBF (mL/min/100g)	44.23 ± 15.87	28.17 ± 17.76	29.47 ± 16.82	23.05 ± 11.24	0.2611	0.6155	0.0714	0.9978	0.8800	0.7759
Dif_TBF (mL/min/100g)	37.66 ± 32.92	35.73 ± 46.34	-3.70 ± 7.56	1.14 ± 7.80	0.9992	0.0417	0.0788	0.0168	0.0369	0.9762
TBF ratio	1.74 ± 0.40	3.49 ± 5.72	0.91 ± 0.27	1.10 ± 0.30	0.7069	0.9532	0.9769	0.2378	0.2825	0.9987
PLD=2525ms										
Lesion TBF (mL/min/100g)	103.45 ± 53.17	73.01 ± 47.82	37.29 ± 25.71	30.89 ± 15.99	0.4175	0.0086*	0.0031*	0.1409	0.0548	0.9750
Muscle TBF (mL/min/100g)	57.23 ± 19.23	36.87 ± 22.69	46.21 ± 31.90	30.89 ± 15.12	0.4174	0.8261	0.1811	0.8219	0.9410	0.4526
Dif_TBF (mL/min/100g)	46.22 ± 40.05	36.13 ± 37.66	-8.92 ± 12.51	-0.01 ± 7.61	0.8899	0.0020*	0.0097*	0.0027*	0.0164	0.8500
TBF ratio	1.76 ± 0.57	2.65 ± 3.23	0.84 ± 0.23	1.03 ± 0.24	0.7708	0.7436	0.8463	0.1029	0.1526	0.9936
PLD=3025ms										
Lesion TBF (mL/min/100g)	151.59 ± 121.14	90.58 ± 58.19	47.32 ± 25.07	44.49 ± 24.69	0.2288	0.0097*	0.0067*	0.3518	0.2798	0.9994
Muscle TBF (mL/min/100g)	67.79 ± 19.65	50.26 ± 28.43	53.29 ± 26.12	45.80 ± 24.05	0.6072	0.7253	0.3889	0.9937	0.9792	0.9051
Dif_TBF (mL/min/100g)	83.80 ± 102.21	40.32 ± 34.78	-5.98 ± 14.18	-1.30 ± 12.47	0.2686	0.0026*	0.0038*	0.0990	0.1451	0.9941
TBF ratio	2.04 ± 0.93	1.85 ± 0.73	0.92 ± 0.30	1.01 ± 0.23	0.9182	0.0035*	0.0071*	0.0040*	0.0090*	0.9773

M-B = comparison between the malignant group and hypervascular benign group. M-L = comparison between the malignant group and hypovascular benign group. M-N = comparison between the malignant group and control group. B-L = comparison between the hypervascular benign group and hypovascular benign group. B-N= comparison between the hypervascular benign group and control group. L-N= comparison between the hypovascular benign group and control group. * $p < .0125$ indicates a significant difference between the malignant, hypervascular benign, hypovascular benign and control groups.
