# Supplementary material for: Feasibility of Arterial Spin Labeling Magnetic Resonance Imaging for Musculoskeletal Tumors with Optimized Post-Labeling Delay
Source: Diagnostics (Basel). 2022 Oct 10;12(10):2450. doi: 10.3390/diagnostics12102450 (PMC9600497; doi:10.3390/diagnostics12102450)
Supplement: Supplementary file 1 [file diagnostics-12-02450-s001.zip › Table S5 difference of the TBF of lesions and muscles in the patients without spiral artifacts.pdf]

Table S5. Difference of the TBF of lesions and muscles in the patients without spiral artifacts. Paired t-test for difference of the mean TBF values of lesions and muscles in four groups with different PLD in the patients without spiral artifacts.

	**Malignant**	**Hypervascular Benign**	**Hypovascular Benign**	**Control**
PLD	*p*	*p*	*p*	*p*
1025ms	0.0342*	0.0101*	0.0953	0.6345
1525ms	0.0288*	0.0159*	0.1148	0.9384
2025ms	0.0628	0.0495*	0.1558	0.6369
2525ms	0.0613	0.0206*	0.0505	0.9975
3025ms	0.1407	0.0084*	0.2153	0.7358

* $p < .05$ indicates a significant difference between the mean TBF values of lesions and muscles.
